# The Transorbital Approach, A Game-Changer in Neurosurgery: A Guide to Safe and Reliable Surgery Based on Anatomical Principles

**DOI:** 10.3390/jcm12206484

**Published:** 2023-10-12

**Authors:** Matteo de Notaris, Matteo Sacco, Francesco Corrivetti, Michele Grasso, Sergio Corvino, Amedeo Piazza, Doo-Sik Kong, Giorgio Iaconetta

**Affiliations:** 1 Laboratory of Neuroanatomy, EBRIS Foundation, European Biomedical Research Institute of Salerno, 84125 Salerno, Italy; 2 Department of Neuroscience, Neurosurgery Operative Unit, “San Pio” Hospital, 82100 Benevento, Italy; 3 Department of Neurosurgery, University of Foggia, 71122 Foggia, Italy; 4 Department of Surgery, Otorhinolaryngology Operative Unit, “San Pio” Hospital, 82100 Benevento, Italy; 5 Department of Neurological Sciences, Division of Neurosurgery, Università degli Studi di Napoli Federico II, 80055 Naples, Italy; 6 Department of Neurosurgery, Sapienza University, 00185 Rome, Italy; 7 Department of Neurosurgery, Samsung Medical Center, School of Medicine, Sungkyunkwan University, Seoul 06531, Republic of Korea; 8 Unit of Neurosurgery, University Hospital San Giovanni di Dio e Ruggi d’Aragona, University of Salerno, 84084 Salerno, Italy

**Keywords:** transorbital surgery, neuroendoscopy, neuroanatomy, skull base surgery

## Abstract

During the last few years, the superior eyelid endoscopic transorbital approach has been proposed as a new minimally invasive pathway to access skull base lesions, mostly in ophthalmologic, otolaryngologic, and maxillofacial surgeries. However, most neurosurgeons performing minimally invasive endoscopic neurosurgery do not usually employ the orbit as a surgical corridor. The authors undertook this technical and anatomical study to contribute a neurosurgical perspective, exploring the different possibilities of this novel route. Ten dissections were performed on ten formalin-fixed specimens to further refine the transorbital technique. As part of the study, the authors also report an illustrative transorbital surgery case to further detail key surgical landmarks. Herein, we would like to discuss equipment, key anatomical landmarks, and surgical skills and stress the steps and details to ensure a safe and successful procedure. We believe it could be critical to promote and encourage the neurosurgical community to overcome difficulties and ensure a successful surgery by following these key recommendations.

## 1. Introduction

**Figure 1:**
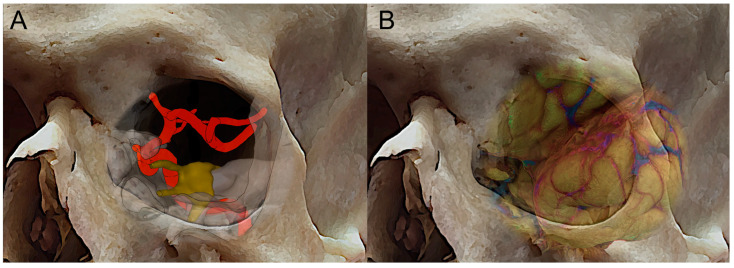
Three-dimensional skull reconstruction of the right orbit with superposition of the course of the internal carotid artey (**A**), the fronto-temporal opercula, and the sylvian fissure (**B**).

During the last few years, neurosurgery has recently been enhanced by a variety of minimally invasive endoscopic procedures to access the ventral skull base. With the advent of transorbital neuroendoscopic surgery (TONES) [1], new modular pathways have been developed for accessing the skull base from the orbit ventrally. This was due to the gradual refinement of endoscopic techniques and anatomical research in minimally invasive skull base surgery. As a matter of fact, anatomic studies have played an important role in the development of transorbital endoscopic approaches, thus providing insights regarding the anatomy [2,3,4,5,6] of the orbit and beyond, such as the paramedian aspect of the anterior and middle cranial fossae and the safest interdural pathway to reach the cavernous sinus laterally to the internal carotid artery (ICA) [7,8] (Figure 1A,B). These studies have allowed surgeons to better understand the relationships between these structures and to identify the safest and most effective approaches for accessing them [4,9,10,11]. In addition, advances in imaging technology, such as computed tomography (CT) and magnetic resonance imaging (MRI), have enabled surgeons to create detailed 3-D models of the ventral perspective of the skull base, which can be used to plan and guide transorbital endoscopic surgeries [12]. Overall, anatomic studies have been instrumental in the development of transorbital endoscopic approaches, helping to make these procedures safer, more precise, and more effective [13,14]. 

Furthermore, transorbital endoscopic approaches have been demonstrated to be feasible for the resection of certain types of tumors, such as trigeminal schwannomas and spheno-orbital meningiomas, with encouraging results [15,16,17]. However, the decision to use a transorbital endoscopic approach must be made on a case-by-case basis by a multidisciplinary team of specialists involving neurosurgeons, maxillofacial surgeons, ophthalmologists, and ENT surgeons [18,19]. 

On the other side, transcranial approaches for paramedian skull base lesions often involve extensive surgeries, which can be associated with significant morbidity and prolonged recovery times. Transorbital endoscopic approaches offer a less invasive alternative, with potential advantages such as shorter hospital stays and faster recovery times [20,21].

Another important thing to consider is that, as recently demonstrated, having experience with endonasal techniques can be valuable for the development of transorbital techniques. Indeed, both endonasal and transorbital approaches involve accessing the ventral skull base. As a matter of fact, experience with endonasal techniques may provide valuable insight into navigating the complex anatomy in the skull base region and identifying potential risks and complications associated with accessing this area. The skills and knowledge developed through endonasal techniques, such as using endoscopes and the ergonomy required to manipulate instruments, may also be applicable to transorbital techniques.

In addition, transorbital and endonasal approaches can be used in combination as part of a multiportal approach to access, simultaneously or not, different median and paramedian areas of the skull base [22,23,24], starting from a key anatomical paradigm: the endonasal approach involves accessing the midline skull base, while the transorbital approach involves accessing the paramedian areas laterally to the parasellar and paraclival segments of the internal carotid artery (Figure 1A) [25]. By combining these approaches in a multiportal approach, surgeons can access a wider range of skull base regions, including areas that may be difficult to reach using a single route [23].

The purpose of this study is to present step-by-step maneuvers that should be observed and accomplished before surgery begins from our 360-degree experience both in anatomy and in clinical settings (endonasal and transorbital surgery). These steps might simplify the often-arduous initial period for beginners, helping them progress more quickly through the steep learning curve. Finally, surgical technique and anatomical studies can provide valuable insights into the potential uses and limitations of the transorbital route. 

This specific knowledge can help surgeons plan and perform procedures using reliable landmarks, reducing the risk of complications and improving patient outcomes.

## 2. Materials and Methods

Anatomical dissections were performed at the Laboratory of Neuroscience, EBRIS, in Salerno (Italy). Ten adult cadaveric embalmed and injected specimens were accessed. Each cadaver head underwent a bilateral superior eyelid transorbital endoscopic approach (TOA). The initial skin and bone step dissections were run under exoscopic visualization for illustrative purposes (Karl Storz) and then continued under endoscopic visualization by means of a rigid 4-mm-diameter endoscope, 18 cm in length, with 0° and 30° rod lenses (Karl Storz GmbH, Tuttlingen, Germany). The common carotid arteries were isolated, cannulated, and injected with red latex. The authors also performed a retrospective review of key and exemplificative transorbital surgery performed by the senior authors to provide specific transorbital techniques applied to an illustrative case.

## 3. Results

### Step-by-Step Paradigm

*1.*  *The endoscopic equipment*

Endonasal and transorbital approaches share almost the same equipment. While there may be some overlap in the instruments and equipment used in these approaches, they are not identical. Similarly, the endoscopic instrumentation used in the endonasal approach may also include a camera head, instruments such as suction devices, dissectors, and drills, and a neuronavigator.

**Figure 2:**
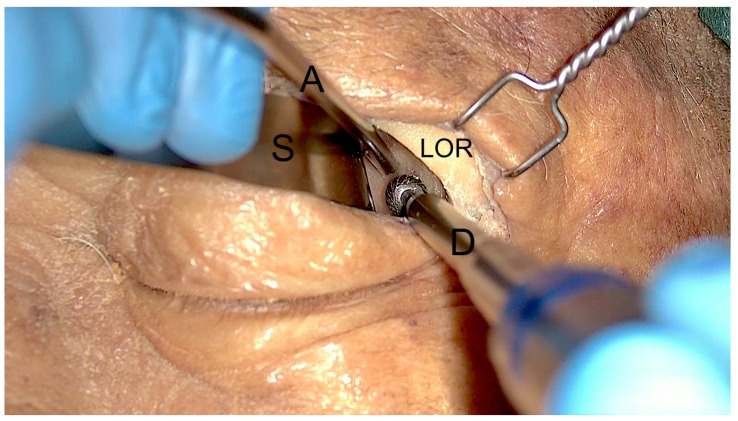
Exoscopic visualization during the first steps of the surgical approach. After superior eyelid incision and subcoutaneous dissection, the lateral orbital rim (LOR) is identified, and a malleable spatula (S) is used to displace medially the orbital content. Two instruments, a low-profile high-speed drill (D) and an aspirator (A), can be inserted inside the surgical corridor along the lateral orbital wall.

The endoscopes designed for transorbital surgery are the same as those for endonasal procedures. In our institution, 4-mm-diameter endoscopes, 18 or 30 cm long, with 0 or 30° angled lenses, are used. The 0 degree scope is used at the beginning of the procedure, while 30 degree scopes are usually used at the end of lesion dissection, either for completing the surgical resection or for inspecting the most hidden and lateral aspects of the surgical field. As for the endonasal procedure, a very useful tip is to use the external sheath connected to a manual or automated irrigation system in which the endoscope is inserted to wash the lens when inside the operating field, which renders the procedure clear and dynamic by avoiding frequent in and out movements over the skin incision and keeping the endoscope lens cleaned. In cases where the exoscope is unavailable, the camera head can also be used alone (without the endoscope connected) as a kind of “external eye” at the beginning of the procedure. In such cases, the “external” visual assistance allowed significant increases in maneuverability by eliminating the space occupied by the endoscope. Indeed, at the beginning of the procedure, the space necessary for carrying out the surgical approach is very limited, and the endoscopes, due to their limited field of view and short focal distances, have various limitations during such steps, meaning that they must be placed within the surgical field with the shaft reducing the available working space and thereby reducing maneuverability. To overcome such limitations, the introduction of a 3-D exoscope system offers new possibilities in visualization and ergonomics specifically exploitable for TOA (Figure 2). 

Once the dissection proceeds medially and deeply and the great sphenoid wing (GSW) is partially removed, the endoscopic visual assistance showed better surgical exposure with increased magnification and illumination potentials, in our experience.

*2.*  *Drills*

The surgical drill used during transorbital procedures should have some specific characteristics to ensure optimal performance and safety. Here are some of the key features: 

*Low profile:* As in the endonasal procedure, the drill should be designed with a low profile to allow easy access to the surgical site without obstructing the surgeon’s view.

*Cut burr:* This type of burr is especially useful in transorbital surgery, where precision and control are crucial, particularly at the beginning of the procedure (4 mm to 5 mm). It can cut through bone with minimal pressure, requiring a large amount of bone to rapidly enlarge the surgical field. 

*Diamond burr:* In addition to the cut burr, the drill may also have a diamond burr (4 mm to 5 mm). Diamond burrs are coated with tiny diamond particles that can grind away bone and other hard tissues with exceptional precision (Figure 2). In addition to their cutting ability, diamond burrs also have the ability to cauterize blood vessels to achieve rapid hemostasis. This can help to reduce bleeding and promote better hemostasis during the final step of the drilling (spongiosum bone) or when the grater sphenoid wing (which represents the first anatomical bony “barrier”) is infiltrated by the lesion (i.e., spheno-orbital meningiomas, chordomas). Overall, diamond burrs are a valuable tool for surgeons performing transorbital procedures, as they can help to improve the endoscopic visualization of the surgical field by reducing the bleeding.

*3.*  *Navigation system*

**Figure 3:**
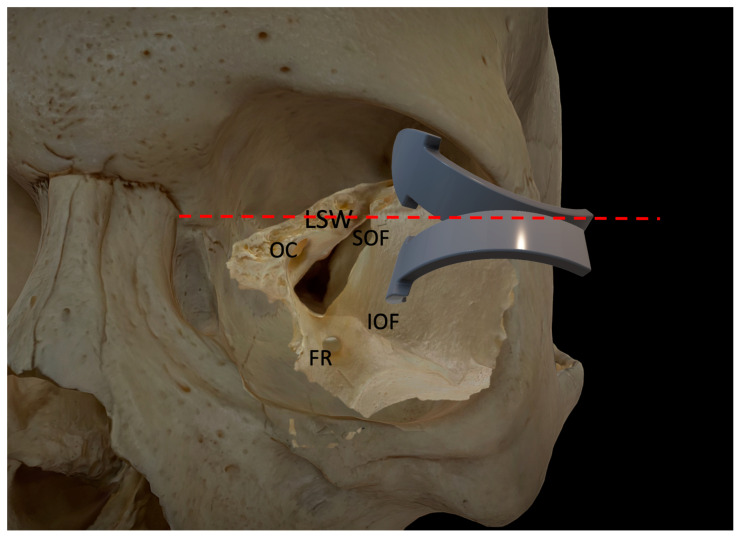
The picture illustrates the two different angles of attack for anterior and middle cranial fossa access. The red dotted line, orthogonal to the fronto-zygomatic suture, represents an imaginary limit between the lateral orbital wall and the orbital roof. FR: foramen rotundum; IOF: inferior orbital fissure; LSW: lesser sphenoid wing; OC: optic canal; SOF: superior orbital fissure.

Neuronavigation is as essential for the transorbital approach as any other skull base surgery. Preoperative imaging, such as CT and MRI scans, allows for the creation of a 3-D roadmap of the patient’s anatomy. During surgery, the surgeon uses the navigational probe to track their position in relation to the patient’s anatomy in real-time. In addition to improving accuracy and reducing the risk of complications, navigation technology can also help reduce the time required for surgery. Overall, navigation is an important tool for transorbital skull base surgery, especially at the beginning of the procedure to localize the first two main landmarks: the superior (SOF) and inferior orbital fissures (IOF). Other important landmarks to localize at the beginning to check the direction of the approach are the position of the optic canal, the clinoidal process, and the lesser wing of the sphenoid in order to understand the transition of the angle of attack from the middle temporal fossa (caudally) to the anterior cranial fossa (cranially) (Figure 3). In our case series, it met our main goal of significantly limiting device cutaneous displacement near the orbit without invasiveness and postoperative discomfort. Finally, the use of a navigation system seems mandatory when transorbital and endonasal approaches are used in combination as part of a multiportal approach in order to plan and recognize intraoperatively the connection of skull base areas between the two routes.

*4.*  *Operating setup and patient position*

The operating setup and patient position for transorbital surgery are critical to ensuring safety and accuracy during the procedure. The patient is positioned supine on the operating table with their head turned 15 degrees, slightly away from the side of the operation. The head is then secured with a Mayfield skull clamp to provide stability and allow neuronavigation. Regarding the position of the surgical team, in our experience, the first surgeon is positioned at the side of the patient, while the assistant may be positioned ahead of the first surgeon, as in endonasal procedures. This allows for the assistant to provide additional support to hold the endoscope and suction, as well as assist with instruments as needed. The position of the nurse during the procedure may also vary depending on the specific shape of the operating room. However, it is common for the nurse to be positioned in front of the first surgeon, providing additional support and assisting with any needs or requests from the surgical team. 

**Figure 4:**
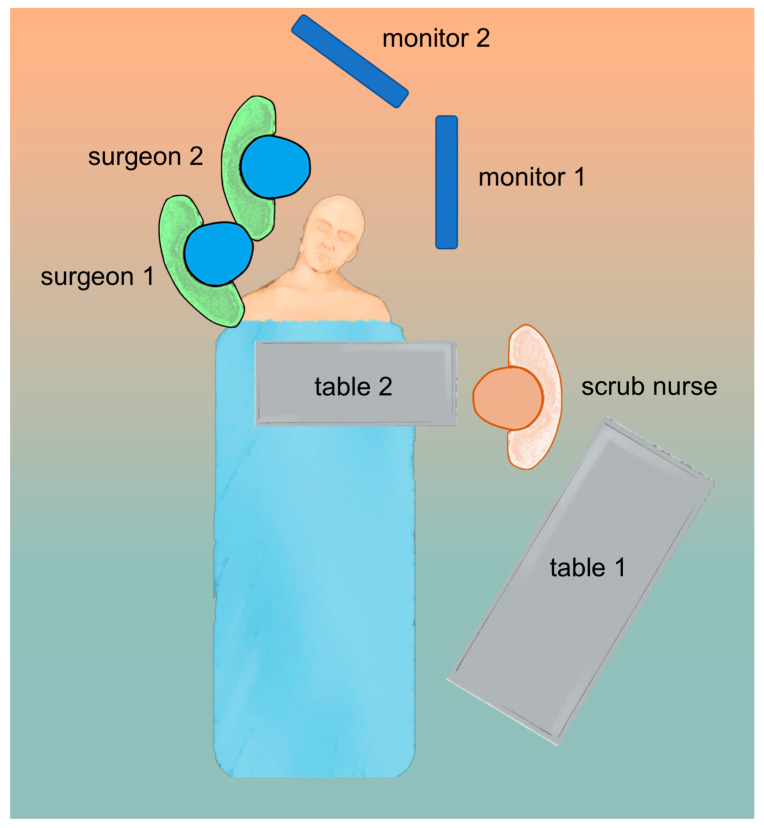
Operative room setup for endoscopic transorbital surgery.

The monitor screens used by the first and second surgeons should ideally be placed in front of them at a comfortable viewing distance and angle. This is important for several reasons: it ensures that they have a clear and unobstructed view of the surgical site; this way, they can maintain a comfortable and ergonomic posture throughout the procedure. This can help to reduce fatigue and improve collaboration between the first and second surgeons, as they can easily communicate and coordinate their actions by referring to the same visual information. The position of the surgical team when a multiportal endonasal and transorbital approach is performed has already been discussed and depicted elsewhere [26] (Figure 4).

*5.*  *Ergonomics—build up your “triangle”*

**Figure 5:**
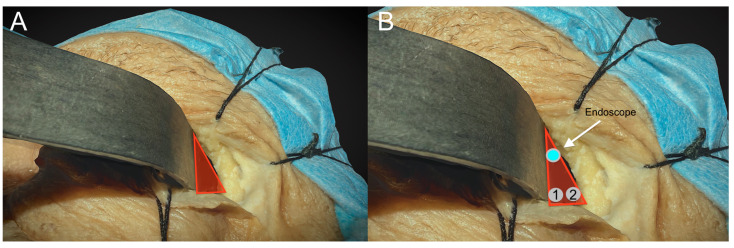
The picture shows the anatomical boundaries of the triangular port. (**A**). The lateral margin is represented by the lateral orbital rim, the medial margin by the retractor itself, and the base is the lateral aspect of the upper eyelid crease. (**B**). The places of insertion of the endoscopic tip are on top, and the two additional instruments are below.

In order to perform a successful and safe surgery, degrees of freedom, angles of vision, ergonomics, and instrument positioning are all of paramount importance. The instruments must be positioned correctly during surgery to avoid instrument collisions and obstructing the vision, exactly as during an endonasal procedure. Indeed, the maneuverability of the endoscope itself can be limited by the crowded transorbital entry site, thus increasing the risk of interference between instruments. As a result of these challenges, ergonomics has been taught and practiced in the anatomical lab, as it happened for transsphenoidal approaches, to provide a clear method to ensure the correct position of the instruments during the surgical approach [3]. After the skin incision at the upper eyelid crease and the progressive medial orbital retraction, the endoscope is inserted into a “virtual space” that is gradually enlarged by drilling the GSW. Due to their limited field of view and short focal distances, endoscopes must be placed within the surgical field, with the shaft reducing the available working area and limiting maneuverability. To solve this issue, the surgeon must construct a triangle-shaped operative area as follows: the lateral margin is represented by the lateral orbital rim, the medial margin by the retractor itself, and the base is the lateral aspect of the upper eyelid crease (Figure 5A).

*6.*  *Evolving from endonasal to transorbital*

Neurosurgeons with experience in neuroendoscopy, particularly endoscopic endonasal surgery, may have a solid foundation to expand their expertise to transorbital surgery. They would already possess knowledge of key endoscopic principles, instrument handling, and navigation within complex skull base regions. Indeed, transorbital surgery can be considered a subset of endoscopic neurosurgery. It requires a similar skill set and familiarity with endoscopic techniques, instrumentation, and anatomical knowledge.

As a matter of fact, equipment and ergonomics for the transorbital approach come directly from the EEA, as both procedures involve advanced endoscopic techniques. Concerning the visualization systems, high-definition monitors and HD or 4K camera systems are used in both transorbital and endonasal surgeries. These systems provide surgeons with a clear view of the surgical field and allow for safe surgery. Similar to EEAs, as the corridor is long and narrow, both procedures require an endoscope for visualization. The endoscope can also be held by an external arm or a second surgeon.

While there are similarities in the equipment and ergonomics, it is worth noting that the transorbital approach may require specific instruments and equipment designed for orbital access and manipulation. Indeed, the main difference among surgical instruments for transorbital surgery is the orbital retractor (Figure 5B). There are different types of malleable or rigid orbital retractors that must be used during the initial step of the approach. For the first cases, we suggest using semi-rigid retractors. These retractors are designed to hold the orbital tissues and maintain the desired exposure without the need for constant manual holding. They come in various sizes and designs that can be adjusted and secured in place. It is important to note that the specific choice of orbital retractors may vary among surgeons and institutions, and there may be other specialized materials utilized for retraction during transorbital procedures, such as protective silastic or silicone sheets. The selection of appropriate retractors depends on the surgeon’s experience and preference.

*7.*  *Key anatomical transorbital principles to access the middle cranial fossa*

According to previously published studies [3,7,25], the skin incision was made through the superior eyelid crease and the orbicularis oculi muscle, and a skin-muscle flap was raised superiorly and inferiorly until the lateral orbital rim was clearly identified. 

**Figure 6:**
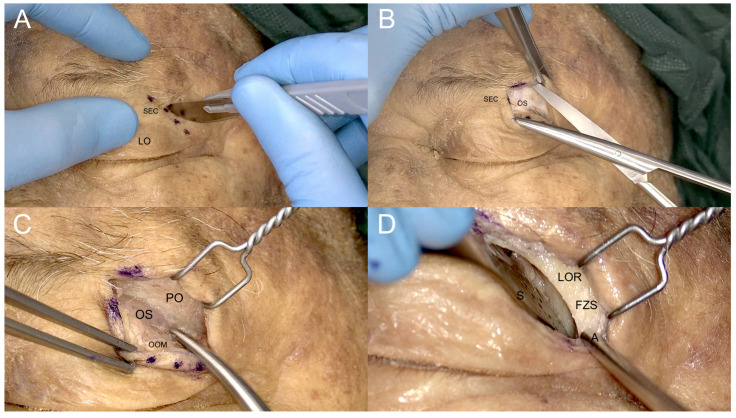
Steps of the superior eyelid transorbital approach. (**A**). Skin incision along the superior eyelid crease (SEC). (**B**). Subcutaneous dissection preserving the optic septum (OS). (**C**). Exposure of the periorbita (PO) of the lateral orbital rim (LOR), with the orbicularis oculi muscle (OOM) separated to preserve the OS underneath. (**D**). Removing the PO and exposing the LOR and the fronto-zygomatic suture (FZS); S: malleable spatula; A: aspirator.

The periosteal layer was then exposed, and a subperiosteal/subperiorbital dissection plane was found and followed. Dissection proceeded using this plane caudally until the IOF first and then the SOF were reached. This way, it was possible to expose a triangular bony area between both fissures that corresponds to the ventral aspect of the GSW. Afterwards, a malleable retractor was then gently introduced to protect the periorbita, displacing the orbital content infero-medially for about 1 cm from the fronto-zygomatic suture and thus creating room for the next surgical steps (Figure 6).

The superior orbital fissure was then protected, and the drilling of the greater sphenoid wing started from laterally to medially. At this point, it was mandatory to first expose the temporal fossa to gain room for further medial progressive dissection. Once the deep temporal muscle fascia is reached, the drilling can turn medially to further remove the medial part of the GSW and gain retrobulbar space. 

The “central core” of the transorbital approach is represented by the exposure of the middle cranial fossa; the other possibilities of this very versatile technique are discussed elsewhere [10].

**Figure 7:**
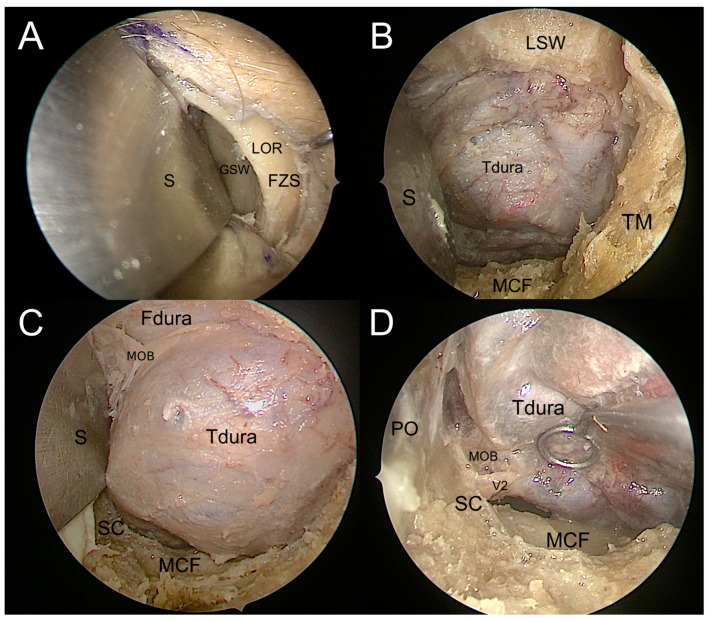
Endoscopic visualization of the transorbital corridor. (**A**). The greater sphenoid wing (GSW) is exposed after identification of the lateral orbital rim (LOR) and frontozygomatic suture (FZS) and subperiosteal dissection of the lateral orbital wall. A malleable spatula (S) is used to retract the orbit. (**B**). Drilling the GSW exposes the dura mater of the temporal lobe (Tdura) in the depth of the surgical field, the temporalis fossa (TF) on the lateral side, the lesser sphenoid wing (LSW) above, and the floor of the middle cranial fossa (MCF) below. (**C**). Drilling the LSW exposes the frontal lobe dura (Fdura) and the meningo-orbital band (MOB) in between; the sagittal crest (SC) is visible in the inferomedial portion of the surgical field. (**D**). Temporal lobe retraction allows for the identification of the maxillary nerve (V2) exiting from the foramen rotundum posterior to the SC; the periorbita (PO) and the MCF can be seen on the medial and inferior sides of the surgical field, respectively.

**Figure 8:**
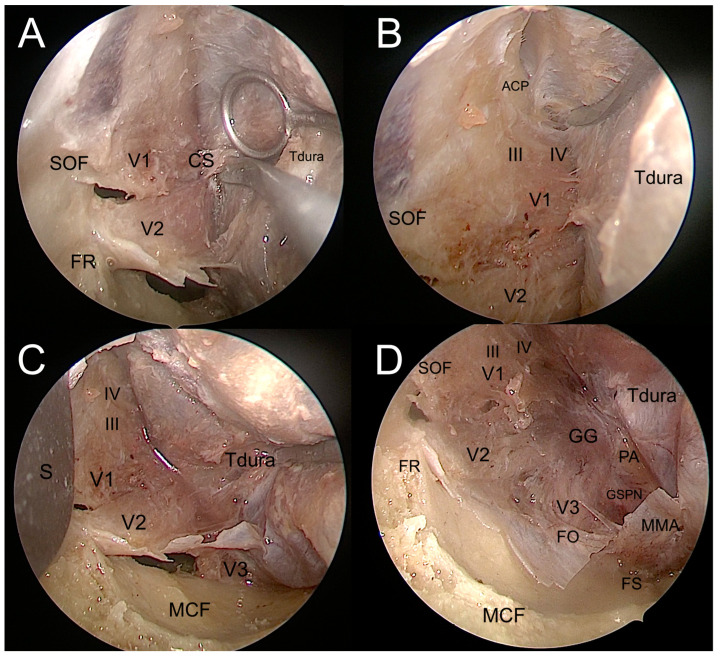
Interdural dissection of the cavernous sinus. (**A**). Exposure of the lateral wall of the cavernous sinus. (**B**). Exposure of the anterior clinoidal process in the upper portion of the surgical field. (**C**). Exposure of the maxillary and mandibulary nerves in the inferior portion of the surgical field. (**D**). Exposure of the entire lateral wall of the cavernous sinus, up to the gasserian ganglion and lateral portion of the middle cranial fossa. ACP: anterior clinoidal process; CS: cavernous sinus; FO: foramen ovale; FR: foramen rotundum; FS: foramen spinosum; III: oculomotor nerve; IV: troclear nerve; GG: gasserian ganglion; GSPN: greater superficial petrosal nerve; MCF: middle cranial fossa; MMA: middle meningeal artery; S: spatula; PA: petrous apex; SOF: superior orbital fissure; Tdura: temporal dura; V1: ophthalmic nerve; V2: maxillary nerve; V3: mandibulary nerve.

By using this straightforward drilling, it gives access to the middle cranial fossa and exposes the dura mater of the temporal pole. While the bone drilling proceeded caudally until the floor of the middle cranial fossa (MCF), the progressive resection of the lesser sphenoid wing allowed further exposure of the dura mater temporal pole. At this point, the medial limit of bone drilling is represented by the most medial portion of the GSW, where the GSW turns progressively from a coronal to a sagittal plane, forming a triangular bony structure shaping dorsally, named the “sagittal crest” [7]. This crest separates the medial temporal dura from the postero-lateral periorbital layer. The sagittal crest was then meticulously drilled until the anterior aspect of the foramen rotundum was encountered, opening the gate to perform the interdural dissection of the cavernous sinus (CS) through the meningo-orbital band [8,27]. Then, the horizontal part of the MOB was cut, exposing the roof of the SOF corresponding to the base of the anterior clinoidal process (ACP) (Figure 7). At this point, the main landmarks over the middle cranial fossa come into view: the foramen rotundum, the foramen ovale, the foramen spinosum, and the middle meningeal artery. While proceeding with the dissection medially and posteriorly to the FO, direct ventral interdural access to the cavernous sinus is achieved (Figure 8).

*8.*  *Illustrative clinical case (the best case to start)*

The case is represented by a 55-year-old male with a history of tobacco smoking (20 packs/year). He was referred for diagnostic endoscopic endonasal endoscopy by his pneumologist during the follow-up of chronic bronchitis, as the patient complained of weight loss and intermittent difficulty swallowing, with associated dysgeusia (described by the patient as a persistent sensation of sour, bitter, and metal taste in the mouth) and exophthalmos. This was associated with progressive left diplopia and blurred vision in the left eye. On admission to our center, magnetic resonance imaging (MRI) of the nasopharynx (NP) and the whole neck region and chest computed tomography (CT) were performed. A brain and neck MRI and CT scan revealed that the primary pharyngeal tumor infiltrated the left paramedian skull base and temporal pole without metastasizing to the upper neck lymph nodes.

**Figure 9:**
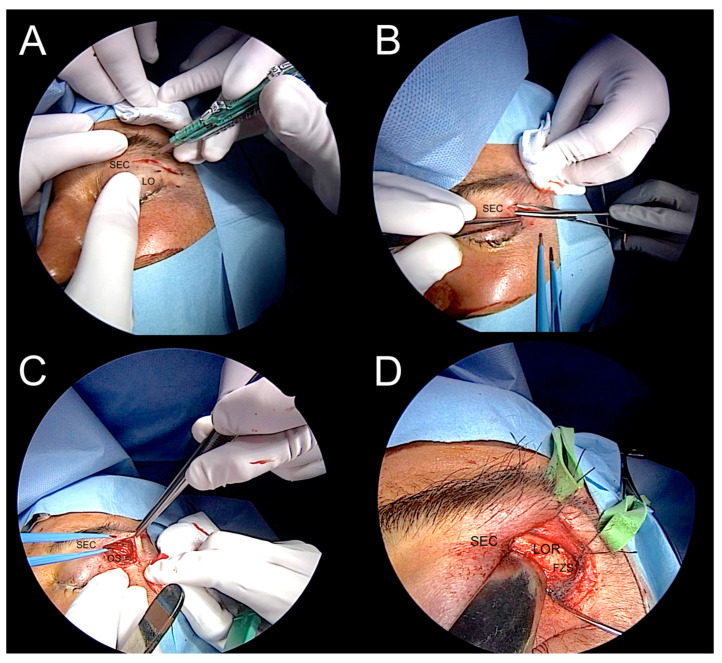
The four steps of transorbital access: (**A**). skin insicion; (**B**,**C**). subcoutaneous dissection; and (**D**). exposure of the lateral orbital rim (LOR). FZS: frontozygomatic suture; LOR: lateral orbital rim; OS: orbital septum; SEC: superior eyelid crease.

**Figure 10:**
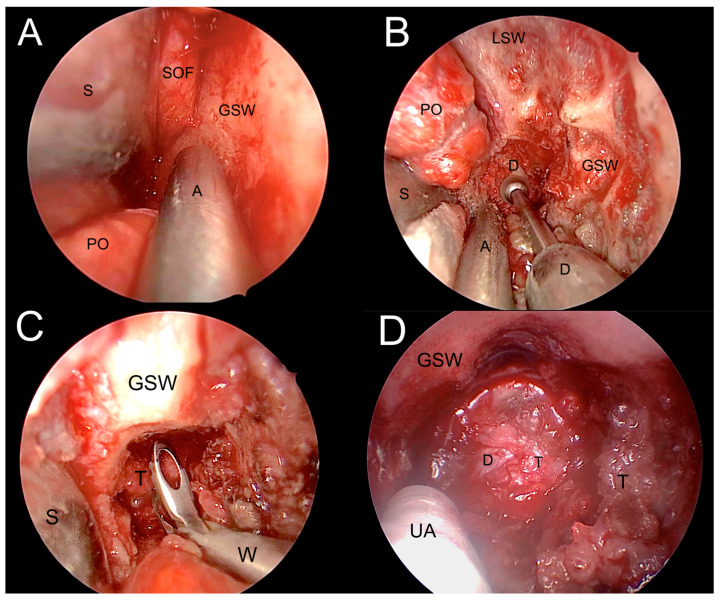
Transorbital corridor and tumor resection. (**A**). Exposure of the lateral orbital wall. (**B**). Drilling the greater sphenoid wing (GSW) to access the middle cranial fossa. (**C**). Tumor biopsy. (**D**). Tumor debulking. A: aspirator; D: dura meter; GSW: greater sphenoid wing; LSW: lesser sphenoid wing; PO: periorbita; T: tumor; S: spatula; UA: ultrasonic aspirator; W: Weil nasal forceps.

A left endoscopic transorbital decompression and biopsy of the lesion were performed using image guidance by the senior author (MdN) (Figure 9 and Figure 10). The procedure revealed an abnormal appearance of greater sphenoid bone infiltration and meningeal granular tissue, also enhanced by contact endoscopy. A biopsy of the infiltrated bone and dura mater revealed several foci of infiltrating carcinoma consistent with metastatic pharyngeal carcinoma. Pathological examination ultimately confirmed the squamous phenotype of the lesion, as initially suspected.

**Figure 11:**
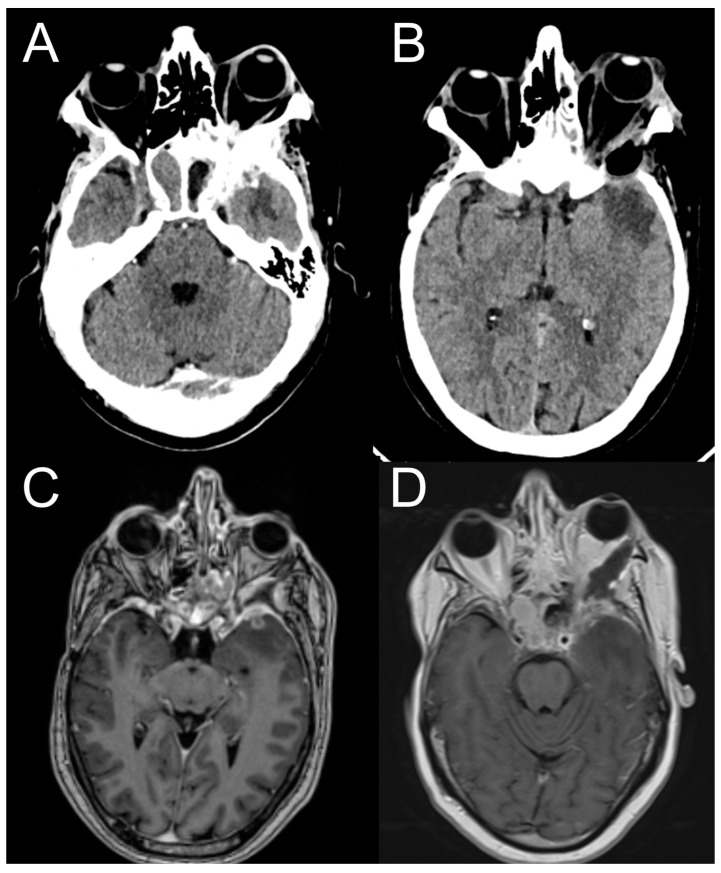
Pre-operative brain CT and MRI (**A**,**C**)show orbital infiltration by the primary tumor involving the temporal dura and the temporal lobe. Post-operative brain CT and MRI (**B**,**D**) show orbital decompression and resection of the brain tumor.

A postoperative TC and MRI scan revealed initial orbital decompression without other complications (Figure 11). The patient was dismissed from our department without a new neurological deficit; the exophthalmos improved one week later, and then he was referred urgently to the oncology department for further evaluation and then adjuvant radiotherapy and chemotherapy.

## 4. Discussion

The transorbital approach provides a direct ventral route to the paramedian skull base, which can achieve a favorable ventral angle of attack for several anterior and middle skull base lesions. As a matter of fact, the ventral perspective of most complex skull base paramedian regions (i.e., the lateral wall of the cavernous sinus, the Meckel’s cave, and the petroclival synchondrosis) allows the surgeon’s eye to be brought close to the target without brain manipulation. Recently, different “extensions” of this ventral paramedian port have been described to reach through a tailored petrous apex drilling in a safe “entry zone” supero-medial to the internal acoustic meatus and without brain retraction or cranial nerve manipulation, the middle tentorial incisura, until the ventral lateral brainstem.

Actually, the transorbital approach is employed for a variety of procedures, including optic nerve decompression [28], biopsy [29], and removal of orbital tumors [30,31], as well as lesions in the anterior and middle skull bases [32] and repair of meningoencephaloceles [33]. It has been shown to be effective and safe in many studies, with low rates of complications and high rates of success [34]. In this context, it can be described as a “game-changer” and revolutionary technique in neurosurgery as a minimally invasive, safe, and reliable technique for multiple reasons: it can lead to reduced morbidity, faster recovery, and shorter hospital stays, avoiding the need for extensive skull base exposure or brain retraction. It provides a direct ventral and paramedian, and intra- and extradural, route to the skull base laterally to the cavernous and clinoidal segments of the internal carotid artery, which can reduce the risk of brain injury, cerebrospinal fluid leak, and infection. This versatility can help surgeons tailor their approach to the specific needs of each patient and surgical target. Furthermore, it can be performed using relatively simple endoscopic equipment, not requiring extensive hospitalization, making it a cost-effective option for many patients.

Finally, this technique is a rapidly evolving field, with ongoing research and development focused on improving surgical techniques and outcomes. New instruments and approaches are being developed to make the transorbital approach even safer and more effective.

## 5. Conclusions

Overall, the transorbital approach represents a major advance in neurosurgery, providing a safe, minimally invasive, and versatile alternative to traditional open cranial approaches. While the technique is still relatively new, its potential benefits for patients and surgeons are significant, and it is likely to become an increasingly important technique in the neurosurgeon’s arsenal.

## Data Availability

The data used in this study are unavailable due to privacy and ethical restriction.
